# Buffalo Medical Library Association

**Published:** 1883-03

**Authors:** A. H. Briggs

**Affiliations:** Secretary


### BUFFALO MEDICAL LIBRARY ASSOCIATION.

*Meeting of Directors.*

OFFICE OF DR. GEO. N. BURWELL,
No. 130 Pearl St., Jan. 13, 1883.

Dr. George N. Burwell, President of the Association, in the Chair.

All the members of the Board were present.

Minutes of former meetings were read and approved.

The committee on by-laws submitted a report, which was adopted.

In conformity with the by-laws, adopted by the Board, the trustees drew lots for their respective terms of office. Doctors Lothrop and Granger drew one year, terms of office to expire at the annual meeting of the Association, to be held in December, 1883, or until their successors are elected.

Doctors Hopkins and Howe drew two years, terms of office to expire at the annual meeting of the Association, to be held in December, 1884, or until their successors are elected.

The committee on location of library submitted a report.

On motion of Dr. Hopkins, the President of the Board was authorized to make a contract with Dr. J. B. Samo, for the location for the Library, in accordance with the following proposition submitted to the Board by Dr. Samo.

BUFFALO, N. Y., Jan. 13, 1883.

I will give my front parlor, carpeted, for the use of the Library Association, with halls and stairs carpeted,—and keep the same in order—furnish a good walnut extension table for the use of the Library, at least until such time as the Association is in funds to buy it or to procure a better one—will also, if desired, place my own medical books, magazines and shelves in the room, and furnish fuel and gas for seventeen ($17.00) dollars per month.

Also in addition to the above for the sum of two hundred and fifty ($250.00) dollars per year I will take charge of the Bureau of Nurses whenever the same shall be established by the Association.

J. B. SAMO.

In accordance with Article VI, Section 1 of the By-Laws, the President appointed the following standing committees.

*Journals*—Drs. Mann, Granger and Howe.

*Books*—Drs. Hauenstein, Mann and Hopkins.

On motion of Dr. Mann, one hundred dollars was appropriated or the use of the Committee on Journals.

On Motion of Dr. Granger, the President appointed Drs. Mann, Hopkins and Abbott a committee to take into consideration the advisability of organizing a Bureau of Nurses to be unders the auspices of the Library Association.

Adjourned subject to the call of the President.

A. H. BRIGGS, *Secretary.*

